# Inequality of opportunity in health service utilization among middle-aged and elderly community-dwelling adults in China

**DOI:** 10.1186/s13690-022-01010-1

**Published:** 2023-01-25

**Authors:** Lin Zhu, Mingyao Peng, Luyan Jiang, Zhonghua Wang

**Affiliations:** 1 School of Health Policy & Management, Nanjing Medical University, 211166 Nanjing, China; 2 Public Health Policy and Management Innovation Research Group, Nanjing Medical University, 211166 Nanjing, China; 3 School of Public Health, Nanjing Medical University, 211166 Nanjing, China

**Keywords:** Inequality of opportunity, Health service utilization, Middle-aged and elderly

## Abstract

**Background:**

The inequality caused by circumstances is known as "inequality of opportunity" (IOp). Many scholars have studied IOp in the health field, but few studies have quantified contributors to the IOp of health service utilization among middle-aged and elderly people. This study measured the IOp of health service utilization and decomposed the contributors to IOp present among Chinese middle-aged and elderly people.

**Methods:**

Data were obtained from the China Health and Retirement Longitudinal Study (CHARLS) in 2013, 2015 and 2018. A mean-based regression method was adopted to measure the IOp of health service utilization. Shapley–Shorrocks decomposition was used to analyze the main contributors to IOp seen among the middle-aged and elderly.

**Results:**

Although the absolute IOp of health service utilization decreased over time, IOp still explains the total inequality to a large extent. The absolute IOp and relative IOp were greatest in the areas of self-treatment and inpatient care utilization, respectively. Shapley decomposition results showed that the out-of-pocket (OOP) ratio contributed most to the IOp of outpatient care utilization; and the residence area highly explains the IOp of inpatient service utilization. Meanwhile, social and economic factors such as work status and income contribute more to the IOp of inpatient care utilization than outpatient and self-treatment.

**Conclusions:**

Strategies aimed at achieving equal opportunities remain necessary to ensure the fairness of health service utilization. Policies and measures should further adjust the medical insurance compensation policies, and pay more attention to the middle-aged and elderly residents in rural areas, optimize health resource allocation, improve the social security systems, and narrow the socioeconomic gap between urban and rural areas in China.

## Background

In addition to improving the health level and efficiency, equality has increasingly become one of the main goals of health reform efforts around the world [1]. Health service utilization is an interactive point between health care users and the health care system, and its equality is the basis for realizing health equity and one of the important indicators for evaluating the effectiveness of health services. With the goal of reducing the inequality of health, equality in access to health services is always high on the policy agendas of countries [2]. One of the priorities of China's new medical reform since 2009 has been to eliminate the inequality of health service utilization [3]. With the universal coverage of social health insurances and health reforms during the past decade, the health service utilization is increasing in China. However, there remain severe challenges to overcoming the inequality of health service utilization in China [4].

Many scholars have studied the inequality of health service utilization. However, most previous studies focused on analyzing the inequality of outcomes of health service utilization [1, 5–7]. Equality of outcomes of health service utilization means that residents with the same needs utilize equivalent health services,however, people often have great differences in personal and environmental characteristics, and only focusing on the inequality of outcomes may lead to inefficient utilization of health services [8]. There is a growing view that public policy design should focus more on the inequality of opportunities rather than the inequality of outcomes [9].

Equality of opportunity can be traced back to a new egalitarian method proposed by the political philosopher Rawls during the last century. This method regards personal responsibility as an important limiting factor of the ethically expected equality, arguing that opportunities should be equally open to all people, regardless of race, religion, or other status, and individuals should also be responsible for their own choices or preferences. Egalitarian theory has gradually developed from equality of results to equality of opportunities [10], in which opportunity refers to "fair competition" or "equal starting points." In 1981, Ronald Dworkin revolutionarily put forward the concept of "resource equality." Resources included aspects of a person's body and biological environment that individuals should not be responsible for [10]. Subsequently, Roemer put forward the theoretical framework of "equal opportunities" [11–13]. In this framework, those factors that an individual can control or be responsible for are called "efforts," while those factors that the individual is not responsible for are called "circumstances." The inequalities caused by these 2 factors should be treated differently [14], specifically, the inequality caused by personal efforts is acceptable, while that caused by circumstances —that is, inequality of opportunity (IOp) [12]—is unacceptable. This framework has facilitated the study of IOp in various fields, gradually extending from economics [15] and education [16] to health [17, 18], health care [19], and even energy consumption [20].

The most common way to understand equal opportunity in health service utilization is from the concept of horizontal equity, which involves providing equal access to health services for those who have the same needs for health services. However, as health service utilization depends upon access conditions and personal preferences, even if access conditions are equal, it is impossible for people with the same demands to use equivalent health services [20]. In view of this, Fleurbaey and Peragine put forward the principle of reward and compensation. Inequality caused by efforts that originated from personal choice or preference is "legal" and does not need compensation, but inequality caused by circumstances is "illegal" and should be compensated for [21]. Therefore, it is quite important to develop health policies for IOp in health service utilization.

Scholars around the world have conducted research on IOp in health care. International scholars have carried out theoretical studies on the applicability and application principles of equal opportunity in the field of health care [22–24]. For example, Harris suggests that everyone has an equal opportunity to benefit from any public health care system and resource allocators should provide people with equal access to health care [25]. Abatemarco et al. redefined the concept of IOp in health care, arguing that IOp in health care should take into account not only the gap between those who have access and those who do not but also the inequality in access conditions among individuals who have access [23]. Some international scholars have confirmed the existence of IOp in the field of health care utilization through empirical studies and found that IOp in health care utilization is often related to socioeconomic factors, such as education and wealth [19, 26, 27]. Most of these studies focused on maternal and child health care. In recent years, Chinese scholars began to pay attention to IOp in health services. Zhang et al. measured the IOp of outpatient and inpatient service utilization of Chinese residents and pointed out that there is a more serious IOp problem in inpatient service utilization [28]. Ma et al. measured and decomposed the inequity gap between urban and rural health care in China [8]. Xiong et al. found that discrepancies in medical service supply, education, and insurance policies were the main reasons for the IOp of urban and rural residents' health service utilization [29]. However, most current studies have focused on general populations or specific health services, and few have paid attention to the IOp of health service utilization among vulnerable Chinese groups.

According to the results of China's seventh census, by the end of 2021, China's population aged ≥ 60 years had reached 267.36 million people, accounting for 18.9% of the country's total population, and those aged ≥ 65 years accounted for 14.2% of the total population [30]. With the aging of the population and the high incidence of chronic diseases among middle-aged and elderly people, the increasing utilization of health services is most significant among the whole population, and the IOp of middle-aged and elderly people's health service utilization is more worthy of attention. Only a few Chinese studies, such as that by Xiong et al. [29], have explored the IOp of middle-aged and elderly people's health service utilization in China. This study pointed out that reforming the health care system from the perspective of equal opportunity may be a more effective way to improve the health care inequality. However, as far as we know, there are few studies quantifying the contribution of impact factors to the IOp of health service utilization among middle-aged and elderly people. In this context, our study sought to understand whether IOp exists in different types of health service utilization among middle-aged and elderly people in China and to what extent IOp is related to its main contributors.

Based on Roemer's "equal opportunity" theory, this study measured the IOp in outpatient, inpatient, and self-treatment utilization among Chinese middle-aged and elderly people. Specifically, we first used a regression method to estimate the related factors of the utilization of different health services among the middle-aged and elderly. Then, an ex-ante method was used to measure the IOp of health service utilization. In addition, Shapley decomposition was used to evaluate the contributions of associated factors to the IOp. Finally, we proposed strategies to alleviate the IOp of health service utilization among middle-aged and elderly people, and provide new evidence for improving health service equity in China.

## Data sources

**Fig. 1:**
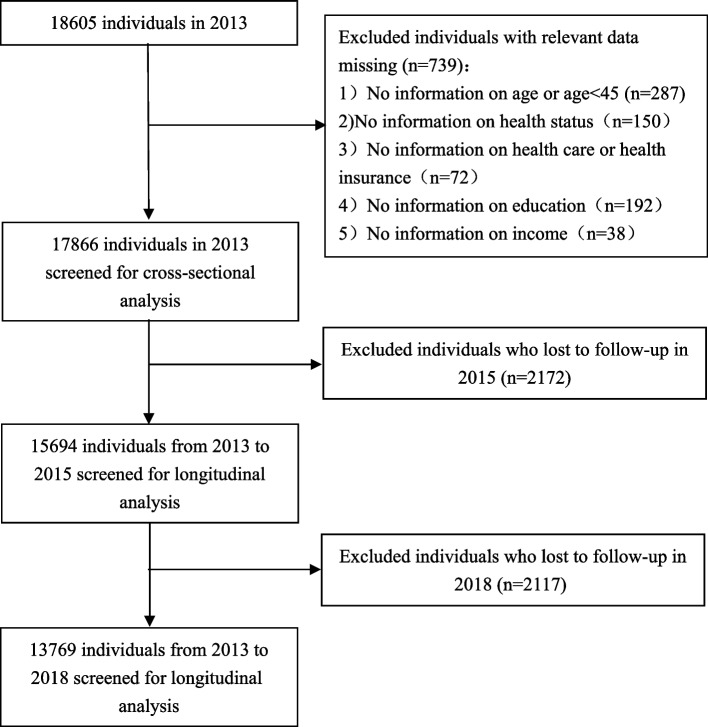
Flowchart of the sample selection process

The study data were obtained from the China Health and Retirement Longitudinal Survey (CHARLS), which were conducted by the National Development Research Institute of Peking University. To ensure the impartiality and representativeness of the sample, CHARLS conducts sampling at 4 levels, which are county (district), village, household, and individual. Probability proportional to size (PPS) is adopted at the county and village levels, and random sampling is adopted at the household and individual levels. CHARLS was conducted in 2011, 2013, 2015, and 2018 in 150 counties and 450 communities (villages) in 28 provinces of China, targeting individuals aged ≥ 45 years and their family members. The survey questionnaire covers personal basic information, family structure and financial support, health status, physical measurement, medical service utilization and medical insurance, work, retirement and pension, income, consumption, and assets. This database is the most representative national database of health and medical service utilization of middle-aged and elderly people in China, which can provide high-quality micro-data support for our research. After excluding samples with missing information on circumstance or effort variables, we constructed a longitudinal dataset of 13,769 consecutive participants surveyed in 2013, 2015, and 2018 to analyze the IOp of outpatient, inpatient, and self-treatment utilization. Figure 1 shows the sample selection process.

## Variable selection

Previous studies have shown that demographic characteristics, education, occupation, insurance status, residential area, etc., are related factors of health service utilization [28, 31–35]. We determined the influencing factors according to existing studies, and then applied the equal opportunity theory to determine the boundary between circumstances and efforts. The IOp was only caused by circumstances according to Roemer's framework [10, 11]. "Effort" is defined as a set of legitimate unequal variables, and individuals should be responsible for it, which can be expressed as medical service demand and preference in the field of health service utilization. Circumstance variables are the illegal sources of unequal utilization of health services, and individuals cannot be responsible for such factors as socioeconomic factors and the impact of medical service supply, so we define them as the "circumstance" [36]. Finally, we collected residence area, medical insurance, socioeconomic factors, subjective and objective health status, health preference, age, and gender details from CHARLS, then further divided the related factors into 3 types of variables: circumstances, efforts, and demographics [12]. Table 1 shows the specific variables and the descriptions.

**Table 1:** Variable definitions

Variables	Definition and Description
**Health Expenditures**	
Outpatient care expenditures	Outpatient care expenditures during the past 1 month (yuan)
Inpatient care expenditures	Inpatient care expenditures during the past 1 year (yuan)
Self-treatment expenditures	Self-Treatment expenditures during the past 1 month (yuan)
**Demographics**	
Age	1 = 45–54 years, 2 = 55–64 years, 3 = 65–74 years, 4 = ≥ 75 years
Gender	1 = male, 0 = female
**Circumstances**	
Urban/rural residence	1 = rural, 0 = urban
Basic medical insurance	1 = yes, 0 = no
Outpatient OOP ratio	Percentage of OOP expenses in total outpatient expenses during the past 1 month (%)
Inpatient OOP ratio	Percentage of OOP expenses in total inpatient expenses during the past 1 year (%)
Self-treatment OOP ratio	Percentage of OOP expenses in total self-treatment expenses during the past 1 month (%)
Distance	Distance from home to the medical facility for the most recent visit (km)
Education	1 = primary education (primary school and below), 2 = secondary education (middle school/high school/vocational school), 3 = higher education (college degree, bachelor’s degree and above)
Work status	0 = no work or retirement, 1 = agricultural work, 2 = other work
Income	Personal annual income (yuan)
**Efforts**	
Self-reported health status	1 = very good, 2 = good, 3 = fair, 4 = poor, 5 = very poor
Chronic disease	Number of chronic diseases
Supplemental medical insurance	1 = yes, 0 = no
Physical examination	1 = yes, 0 = no

*Notes*: *OOP* Out-of-pocket

### Health service utilization

Some researches measure health service utilization by 2-week outpatient rate, hospitalization rate, etc. [3, 37], and many others use health service expenditures [2, 26, 38, 39]. In this study, we measured health service utilization among middle-aged and elderly people by the expenditures of outpatient care, inpatient care, and self-treatment. According to CHARLS, we define outpatient expenses as medical expenses paid for outpatient services in medical institutions in the past month. Inpatient care expenditure refers to the medical expenses paid for hospitalization services in medical institutions in the past year (only including fees paid to the hospital). Self-treatment expenditure includes the medical expenses of self-treatment behaviors such as buying medicines, treating with traditional Chinese herbal medicines or traditional methods, taking health care products or using health care equipment in the past month. All expenditures were converted to the price level in 2018 using a price index. In order to prevent bias due to large variances in expenditure values, we logged all expenditure variables. If individuals spent no money on health service, their expenditure values are set to 0 [8].

### Circumstances

Circumstances are considered "illegal" factors of inequality of health service utilization, that is, the non-need factor of health service utilization, such as medical service supply, medical financing, etc. [29]. Residence area has always been considered an important factor in the inequality of health service utilization due to the great disparity between urban and rural areas in China. Health insurance helps middle-aged and elderly people access and utilize high-quality health services, and the out-of-pocket (OOP) ratio represents the reimbursement level of health insurance, both of which are important circumstance factors. The distance from home to the medical facility is the supplier variable of health services, which can reflect the time and transportation costs of medical treatment among middle-aged and elderly people. Education, work status and income level are used to measure an individual's socio-economic status, which is greatly influenced by the social environment and also included in circumstance factors [12, 40]. Income values from 2013 and 2015 were converted according to the price level in 2018 through the price index.

### Efforts

Effort is a personal choice and individuals should be responsible for their own preferences [11]. Individuals can change their health status through self-efforts, thus affecting the utilization of health services. Previous studies have shown that people with poor health status are more likely to use health services [41]. Therefore, we chose the "self-reported health status" to represent an individual's subjective health status. In addition, CHARLS investigated the prevalence of 14 kinds of chronic diseases, including hypertension, diabetes, chronic obstructive pulmonary disease, and so on. Therefore, we sorted out the number of chronic diseases suffered by middle-aged and elderly people to represent the objective health status. Supplementary medical insurance and physical examination were also selected to represent individual preference variables, and those who purchased supplementary medical insurance and participated in physical examinations may have a greater preference for health service utilization [41].

### Demographics

We included age and gender as the main demographic variables for health service utilization because these 2 predisposing factors are major elements affecting health service utilization and have been analyzed in many previous studies [2, 40, 41]. Furthermore, in the field of health, most regard age and gender as biological factors, and health policies cannot eliminate the influence of age or gender. Therefore, we consider them to be characteristics that are neither under personal control nor required to be compensated for.

## Methods

### Estimation of IOp (mean-based regression)

Following Roemer's framework, personal outcomes or achievements depend on circumstances (personal uncontrollable), efforts (personally responsible), and demographics (including gender and age). Therefore, the health service utilization result ${\mathcal Y}_i$ of middle-aged and elderly people $i$ can be written as a production function of $C_i,E_i,\ and\;D_i$ through the following equation:

1 $${\mathcal{Y}}_{i}=f\left({C}_{i}, {E}_{i},{D}_{i},{\mu }_{i}\right)$$

where ${\mathcal{Y}}_{i}$ represents the utilization of health services; $C_i$ and $E_i$ are factors of circumstances and efforts, respectively; $D_{i}$ represents personal demographics; and ${\mu }_{i}$ is unobserved error terms.

In previous studies, many researchers discussed parametric and non-parametric approaches to evaluate IOp [9, 15, 42]. Both parametric and non-parametric methods aim to distinguish inequalities caused by circumstances from those caused by efforts [14]. Compared to a non-parametric method, a parametric estimation method has 2 advantages: it is not limited by multiple dimensions, especially for a rich set of circumstances, and it can calculate the contribution of each factor to inequality through Shapley decomposition. Therefore, we adopted the direct ex-ante parameter method proposed by Ferreira and Gignoux to estimate the determining equation of health service utilization by mean-based regression [9], so the determining equation for health service utilization can be written as a linear model as follows:

2 $${\mathcal{Y}}_{i}= {\alpha C}_{i}+ {\beta \mathrm{\rm E}}_{i}+ {\gamma D}_{i}+ {\mu }_{i}$$

The ex-ante approach assumes that, if all middle-aged and elderly people are facing the same opportunities before making efforts and making use of health services, then equality of opportunity exists [43]. Roemer's definition of IOp requires that all inequality due to efforts should be eliminated, leaving only that caused by circumstances. However, the effort is, in some sense, determined by the circumstance, and can be estimated by an auxiliary equation, as follows:

3 $${E}_{i}= \eta + {\lambda C}_{i}+ {e}_{i}$$

where the residual term ${e}_{i}$ is the relative efforts, which represent the efforts after the circumstances impact has been removed.

After organizing Eqs. 2 and 3, the production function of health service utilization can be rewritten using the following equation:

4 $${\mathcal{Y}}_{i}= {\alpha C}_{i}+ {\beta \widehat{e}}_{i}+ {\gamma D}_{i}+ {\mu }_{i}$$

where $\alpha$ encompasses both the direct effect of circumstances on ${\mathcal{Y}}_{i}$ and the indirect effect of circumstances through efforts, while $\beta$ captures the direct contribution of efforts. We used ordinary least squares (OLS) to estimate the health service utilization among middle-aged and elderly people.

In order to measure the IOp, we used an inequality index——Gini coefficient, which is widely used to measure the IOp in many fields such as income and education [43–46]. In this paper, we referred to the research ideas of previous literatures and used the Gini coefficient of the original sample to represent the total inequality. Based on the OLS estimation results, we calculate the Gini index of the predicted value of the regression model to obtain the absolute level of IOp, which can be expressed with the following formula [9], where ${\mathrm{\rm I}}_{0}$ denotes the Gini index and $\widehat{{\mathcal{Y}}_{i}}$ depicts the predicted outcome:

5 $${\uptheta }_{a}= {\mathrm{\rm I}}_{0}\left(\widehat{{\mathcal{Y}}_{i}}\right)$$

If IOp does not exist, the Gini coefficient of the predicted value of the regression model should be the same as that of the original sample. For the convenience of observation and comparison, we calculated the ratio of IOp to total inequality, which can be regarded as relative IOp [47], and expressed by the following formula:

6 $${\uptheta }_{r}= \frac{{\mathrm{\rm I}}_{0} \left(\widehat{{\mathcal{Y}}_{i}}\right)}{{\mathrm{\rm I}}_{0} \left({\mathcal{Y}}_{i}\right)}$$

### Shapley-Shorrocks decomposition

Shorrocks proposed the Shapley decomposition method based on the "Shapley value" to decompose inequality [48]. The regression-based Shapley decomposition method can effectively calculate the contributions of each determinant to the overall inequality. The Shapley decomposition method has 2 advantages, as follows: 1) the Shapley value is additive, so we can calculate the contribution values of sub-variables to get the total contribution of that type of variable to inequality [49], and 2) it is independent of the order of decomposition—that is, the order in which variables change during the decomposition process does not affect the results. Therefore, we used Shapley–Shorrocks decomposition to measure the relative contributions of circumstances (IOp), efforts, and demographics to total inequality.

## Results

### Descriptive statistics

Table 2 shows the statistical description of the sample. From 2013 to 2018, expenditures on the 3 types of health services continued to increase. Specifically, outpatient expenditures increased by 38.24% in 2015 (1388.00; compared to 2013) and 12.10% in 2018 (1,579.00; compared to 2015), while inpatient care expenditures increased by 11.94% (16,560.00; compared to 2013) and 38.99% (27,144.00; compared to 2015) in 2015 and 2018, respectively, while self-treatment expenditures remained stable. In terms of demographic characteristics, the proportion of people aged ≥ 65 years in the whole sample increased gradually, from 29.13% in 2013 to 48.88% in 2018. Among the 13,769 total participants, men accounted for 47.08% (6483) and women accounted for 52.92% (7286).

**Table 2:** Descriptive statistics

Variables	2013			2015			2018		
	Mean/Proportions	Obs.	SD	Mean/Proportions	Obs.	SD	Mean/Proportions	Obs.	SD
**Health Expenditures**									
Outpatient care expenditures (yuan)	857.20	13,769	2147.00	1388.00	13,769	4306.00	1579.00	13,769	5373.00
Inpatient care expenditures (yuan)	14,583.00	13,769	20,169.00	16,560.00	13,769	23,527.00	27,144.00	13,769	65,948.00
Self-treatment expenditures (yuan)	208.90	13,769	589.10	308.90	13,769	907.60	290.20	13,769	774.50
**Demographics**									
Age	2.05	13,769	0.92	2.20	13,769	0.96	2.51	13,769	0.93
1 = 45–54 years (%)	32.30	4448		27.16	3740		14.54	2002	
2 = 55–64 years (%)	38.57	5311		36.20	4985		36.58	5037	
3 = 65–74 years (%)	21.29	2931		25.95	3573		32.75	4509	
4 = ≥ 75 years (%)	7.84	1079		10.58	1471		16.13	2221	
Gender	0.47	13,769	0.50	0.47	13,769	0.50	0.47	13,769	0.50
0 = female (%)	52.92	7286		52.92	7286		52.92	7286	
1 = male (%)	47.08	6483		47.08	6483		47.08	6483	
**Circumstances**									
Urban/rural residence	0.81	13,769	0.39	0.80	13,769	0.40	0.81	13,769	0.39
0 = urban (%)	18.69	2573		19.94	2745		18.60	2561	
1 = rural (%)	81.31	11,196		80.06	11,024		81.40	11,208	
Basic medical insurance	0.94	13,769	0.23	0.90	13,769	0.30	0.95	13,769	0.21
0 = no (%)	5.73	789		10.25	1412		4.70	647	
1 = yes (%)	94.27	12,980		89.75	12,357		95.30	13,122	
OOP ratio									
Outpatient OOP ratio (%)	87.97	1,382	23.61	87.89	1,275	23.70	86.79	1,090	25.13
Inpatient OOP ratio (%)	57.34	427	30.35	60.47	522	30.01	56.72	828	30.40
Self-treatment OOP ratio (%)	96.68	7,265	13.54	96.59	7,074	13.49	95.96	7,941	14.90
Distance (km)	6.84	13,769	50.00	8.82	13,769	62.11	14.50	13,769	117.20
Education	1.35	13,769	0.51	1.35	13,769	0.51	1.38	13,769	0.53
1 = primary education (%)	67.25	9260		66.71	9185		64.05	8819	
2 = secondary education (%)	31.04	4272		31.47	4333		33.84	4660	
3 = higher education (%)	1.71	235		1.82	251		2.11	290	
Work Status	0.85	13,769	0.63	0.97	13,769	0.65	0.73	13,769	0.66
0 = no work or retirement (%)	28.61	3939		22.80	3139		39.49	5438	
1 = agricultural work (%)	57.60	7931		57.32	7892		48.46	6673	
2 = other work (%)	13.79	1899		19.89	2738		12.04	1658	
Income (yuan)	10,608.00	13,769	21,656.00	11,527.00	13,769	36,556.00	15,262.00	13,769	30,155.00
**Efforts**									
Self-reported health status	2.99	13,769	0.95	2.96	13,769	1.01	3.01	13,769	1.01
1 = very good (%)	8.86	1220		11.66	1606		10.81	1489	
2 = good (%)	14.11	1943		12.32	1697		11.86	1633	
3 = fair (%)	51.86	7141		50.34	6931		48.83	6724	
4 = poor (%)	19.75	2720		19.94	2745		22.07	3039	
5 = very poor (%)	5.41	745		5.74	790		6.42	884	
Chronic disease (number)	1.29	13,769	1.37	1.52	13,769	1.49	0.68	13,769	0.99
Supplemental medical insurance	0.11	13,769	0.31	0.04	13,769	0.20	0.07	13,769	0.26
0 = no (%)	89.20	12,282		95.66	13,171		92.51	12,738	
1 = yes (%)	10.80	1487		4.34	598		7.49	1031	
Physical examination	0.34	13,769	0.48	0.40	13,769	0.49	0.46	13,769	0.50
0 = no (%)	65.61	9034		59.86	8242		54.03	7439	
1 = yes (%)	34.39	4735		40.14	5527		45.97	6330	

*Notes*: *Obs* Observations, *SD* Standard deviation, *OOP* Out-of-pocket

Regarding circumstance factors, the number of middle-aged and elderly people with basic medical insurance increased (from 89.75% to 95.30%) during the period of 2013–2018. The OOP ratio of self-treatment was the highest (95.96%–96.68%), followed by that of outpatient (86.79%–87.97%) and inpatient (56.72%–60.47%) expenses. In addition, the vast majority of respondents (67.25%–64.05%) had only received a primary education. Most of the respondents were engaged in agricultural work (48.46%–57.60%) or were unemployed and retired (22.80%–39.49%).

As for effort factors, from 2013 to 2018, the self-reported health status of middle-aged and elderly people showed a slight deterioration (from 2.99 in 2013 to 3.01 in 2018), while their objective health status improved, with a reduction in the number of chronic diseases from 1.29 in 2013 to 0.68 in 2018. The proportion of middle-aged and elderly people having supplementary medical insurance remained low, but the awareness of physical examinations has gradually increased: 34.39% chose to participate in physical examinations in 2013, which increased to 45.97% in 2018.

### Mean-based estimation of IOp

We first conducted a regression analysis on the associated factors of health expenditures (see Table 3). The results showed that elderly individuals aged 65–74 years made less use of outpatient services (*P* < 0.1) and inpatient services (*P* < 0.05) in 2013 than those aged 45–54 years. Male patients' hospitalization expenditures were significantly higher than that of female patients in 2013 (*P* < 0.1) and 2018 (*P* < 0.01), while female patients' self-treatment expenditures were significantly higher than those of male patients in 2015 (*P* < 0.01) and 2018 (*P* < 0.05).

**Table 3:** OLS regression on different health expenditures among middle-aged and elderly people

Health Expenditures (log)	2013			2015			2018		
	Outpatient	Inpatient	Self-treatment	Outpatient	Inpatient	Self-treatment	Outpatient	Inpatient	Self-treatment
**Demographics**									
Age (ref. 45–54 years)									
55–64 years	0.004	-0.067	0.017	-0.011	-0.001	0.005	0.025	0.068	0.007
	(0.13)	(-1.06)	(1.29)	(-0.31)	(-0.02)	(0.36)	(0.58)	(1.11)	(0.46)
65–74 years	-0.059*	-0.133**	0.001	-0.051	-0.049	0.010	-0.045	0.064	-0.010
	(-1.81)	(-2.13)	(0.05)	(-1.46)	(-0.74)	(0.65)	(-1.03)	(0.96)	(-0.62)
≥ 75 years	-0.036	-0.032	-0.018	-0.059*	-0.100	-0.008	-0.047	-0.005	0.011
	(-1.20)	(-0.57)	(-1.40)	(-1.83)	(-1.60)	(-0.60)	(-1.16)	(-0.08)	(0.69)
Male (ref. female)	-0.012	0.085*	-0.018	0.030	0.016	-0.072***	0.049	0.102***	− 0.023**
	(-0.44)	(1.85)	(-1.59)	(1.08)	(0.35)	(-6.08)	(1.64)	(3.01)	(− 2.17)
**Circumstances**									
Rural (ref. urban)	-0.054*	-0.077	-0.069***	-0.091***	-0.021	-0.079***	− 0.085***	− 0.146***	− 0.084***
	(-1.76)	(-1.31)	(-5.03)	(-3.00)	(-0.44)	(-6.05)	(− 2.72)	(− 4.12)	(− 7.33)
Basic medical insurance (ref. no)	-0.010	-0.027	-0.002	-0.043	-0.032	-0.023**	0.006	0.006	0.015
	(-0.41)	(-0.61)	(-0.20)	(-1.60)	(-0.75)	(-2.02)	(0.22)	(0.17)	(1.39)
OOP ratio	− 0.224***	− 0.119***	− 0.095***	− 0.219***	− 0.103**	− 0.082***	− 0.209***	− 0.114***	− 0.125***
	(− 8.72)	(− 2.61)	(− 8.76)	(− 8.07)	(− 2.24)	(− 7.27)	(− 7.15)	(− 3.40)	(− 11.93)
Distance	0.090***	0.183***	0.106***	0.166***	0.055	0.091***	0.141***	0.115***	0.040***
	(3.55)	(4.11)	(9.75)	(6.26)	(1.32)	(8.10)	(4.91)	(3.47)	(3.83)
Education (ref. primary education)									
Secondary education	0.040	0.055	0.005	0.030	0.068	0.022*	0.021	0.013	− 0.022**
	(1.38)	(1.15)	(0.38)	(1.02)	(1.45)	(1.73)	(0.71)	(0.38)	(− 1.98)
Higher education	-0.006	0.013	0.024**	0.021	0.068	0.047***	− 0.026	0.008	− 0.014
	(-0.21)	(0.27)	(2.07)	(0.74)	(1.56)	(3.96)	(− 0.83)	(0.23)	(− 1.23)
Work status (ref. no work or retirement)									
Agricultural work	-0.111***	-0.263***	-0.096***	-0.137***	-0.224***	-0.094***	− 0.148***	− 0.178***	− 0.105***
	(-3.63)	(-5.39)	(-6.92)	(-3.66)	(-4.13)	(-5.88)	(− 4.41)	(− 4.88)	(− 8.31)
Other work	-0.024	-0.096**	-0.059***	-0.028	-0.069	-0.057***	− 0.043	− 0.038	− 0.082***
	(-0.84)	(-1.98)	(-4.52)	(-0.81)	(-1.42)	(-3.93)	(− 1.33)	(− 1.09)	(− 6.84)
Income	0.011	0.061	0.032**	-0.047	0.105**	0.010	0.002	0.039	− 0.007
	(0.35)	(0.97)	(2.46)	(-1.57)	(2.27)	(0.85)	(0.05)	(1.04)	(− 0.62)
**Efforts**									
Self-reported health status (ref. very good)									
Good	-0.007	-0.125	0.042**	0.001	-0.074	0.006	− 0.015	0.053	0.018
	(-0.15)	(-1.62)	(2.43)	(0.03)	(-1.25)	(0.43)	(− 0.33)	(0.92)	(1.19)
Fair	0.012	-0.167	0.182***	-0.079*	-0.184**	-0.009	− 0.086	− 0.003	0.142***
	(0.13)	(-1.18)	(7.74)	(-1.70)	(-2.45)	(-0.50)	(− 1.07)	(− 0.03)	(6.84)
Poor	0.148*	-0.145	0.290***	-0.023	-0.051	-0.000	− 0.026	− 0.042	0.269***
	(1.67)	(-1.00)	(13.51)	(-0.57)	(-0.75)	(-0.01)	(− 0.33)	(− 0.40)	(13.71)
Very poor	0.171***	0.057	0.232***	0.001	-0.065	0.035**	− 0.002	0.058	0.214***
	(2.78)	(0.49)	(14.80)	(0.04)	(-1.13)	(2.57)	(− 0.03)	(0.66)	(14.46)
Chronic disease	0.033	-0.068	0.127***	0.044	-0.022	0.196***	0.105***	0.092***	0.122***
	(1.28)	(-1.51)	(11.07)	(1.62)	(-0.50)	(16.91)	(3.60)	(2.78)	(11.34)
Supplemental medical insurance (ref. no)	0.008	0.062	-0.012	0.054**	0.109**	-0.007	− 0.048	0.031	0.017
	(0.30)	(1.41)	(-1.12)	(2.02)	(2.57)	(-0.63)	(− 1.64)	(0.94)	(1.58)
Physical examination (ref. no)	0.085***	0.100**	0.067***	0.045	0.051	0.073***	0.026	− 0.055	0.035***
	(3.28)	(2.25)	(5.99)	(1.65)	(1.20)	(6.26)	(0.86)	(− 1.62)	(3.22)
Constant	6.886***	10.089***	4.712***	7.879***	9.940***	5.581***	7.598***	9.796***	5.530***
	(18.78)	(17.68)	(29.01)	(27.65)	(27.53)	(34.35)	(20.92)	(25.72)	(40.21)

*Notes: t* statistics are shown in parentheses. Estimates of coefficients are standardized. *** *P* <0.01, ** *P* < 0.05, * *P* <0.1

Among the circumstances, middle-aged and elderly people with lower OOP ratios or who were traveling further to receive medical treatment had higher medical expenses (*P* < 0.01). The absolute regression coefficient of OOP ratio is the largest in outpatient service utilization, indicating that OOP ratio has greater impact on outpatient service utilization than that of hospitalization and self-treatment, Distance had a greater influence on the utilization of inpatient services in 2013, while it was greater on the utilization of outpatient services in 2015 and 2018. Residence, education, and work status were also significant factors affecting the utilization of health services. Respondents who lived in urban areas used more outpatient care services in 2015 and 2018 (*P* < 0.01), more inpatient services in 2018 (*P* < 0.01), and more self-treatment in all waves (*P* < 0.01) compared with rural residents. People with higher education used more self-treatment (*P* < 0.05 in 2013; *P* < 0.01 in 2015). Those who were unemployed and retired also had higher medical expenses (*P* < 0.01). In addition, the results of 2013 and 2015 showed that, the higher the income, the more the hospitalization and self-treatment expenses (*P* < 0.05).

All effort factors except for supplementary medical insurance significantly affected the utilization of self-treatment services among middle-aged and elderly people in 2013, 2015, and 2018. Respondents with worse self-reported health status (*P* < 0.01) or who participated in physical examinations (*P* < 0.01) in 2013 showed more outpatient utilization, and those with more chronic diseases also had significantly increased outpatient (*P* < 0.01) and inpatient (*P* < 0.01) spending in 2018. Supplementary medical insurance had a positive effect on the utilization of outpatient and inpatient services (*P* < 0.05) in 2015.

According to the OLS results of Table 3, Gini index was used to calculate the total inequality and IOp in different health service utilization in Table 4. From 2013 to 2018, the IOp (column a) and total inequality (column b) of the 3 types of health expenditures demonstrated an overall downward trend. Column a shows the absolute value of IOp of each health service; specifically, the IOp of self-treatment utilization was the highest, followed by that of outpatient care, and that of inpatient care is the lowest and declined the most (i.e., by 13.85% from 2013 to 2018). However, as demonstrated in column c, the IOp ratios in inpatient utilization in 2013 (50.18%) and 2015 (39.84%) were the highest. From 2013 to 2018, 36.33%–50.18% of the total inequality in inpatient utilization was attributed to IOp, while 34.06%–36.85% of the same in outpatient care and 31.63%–38.73% in self-treatment expenditures were similarly attributed.

**Table 4:** Total inequality and IOp in health expenditures (Gini index)

Health Expenditures	IOp (a)	Total Inequality (b)	IOp Ratio (%) (c)
**Panel A: 2013**			
Outpatient	0.05105	0.13852	36.85%
Inpatient	0.03896	0.07764	50.18%
Self-treatment	0.07440	0.19211	38.73%
**Panel B: 2015**			
Outpatient	0.04826	0.14126	34.16%
Inpatient	0.03065	0.07694	39.84%
Self-treatment	0.06119	0.19346	31.63%
**Panel C: 2018**			
Outpatient	0.04410	0.12949	34.06%
Inpatient	0.02744	0.07552	36.33%
Self-treatment	0.05958	0.16162	36.86%

### Shapley decomposition

**Fig. 2:**
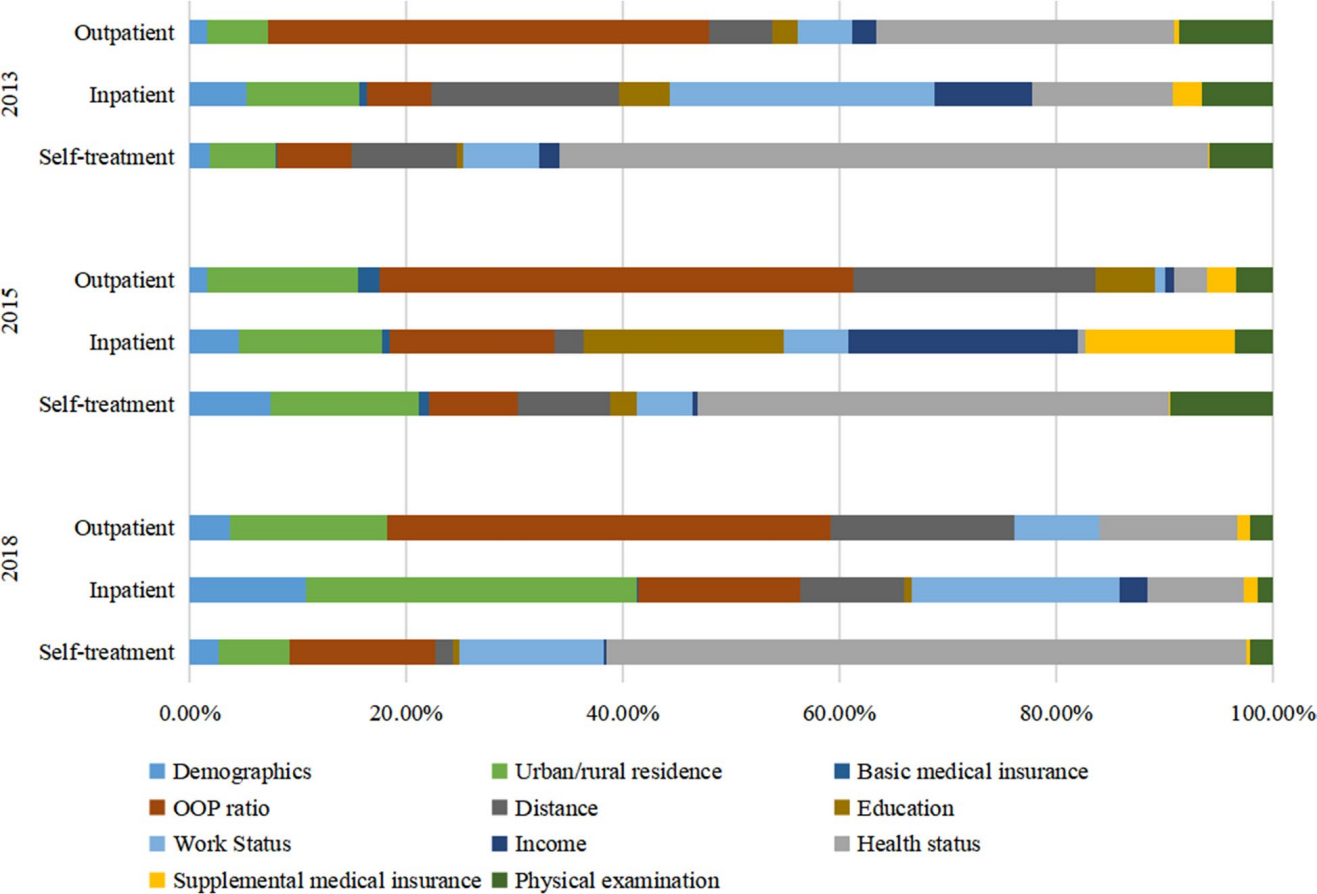
Mean-based Shapley decomposition results

Table 5 and Fig. 2 demonstrate the contributors of the inequality of health service utilization among middle-aged and elderly people. It is worth noting that the contribution of circumstances to the unequal utilization of health services increased during the investigation period, while the contribution of effort factors decreased. Specifically, we found that the contribution of circumstance factors to the total inequality of outpatient and inpatient service utilization was dominant, accounting for 61.77%–89.32% for outpatient care and 72.56%–77.66% for inpatient care, and the contribution of effort factors to the total inequality held for 9.10%–36.61% in outpatient care and 11.58%–22.15% in inpatient care, respectively. However, the situation was reversed in the context of self-treatment utilization, where effort factors contributed the most, with a contribution of 53.10%–65.91%, followed by the contributions of circumstance factors, which ranged from 32.25% in 2013 to 35.80% in 2018. The contributions of demographic factors to outpatient service, inpatient service, and self-treatment utilization were relatively small, being 1.59%–3.69%, 4.54%–10.77%, and 1.85%–7.45%, respectively.

**Table 5:** Mean-based Shapley decomposition results

	2013			2015			2018		
Health Expenditures	Outpatient	Inpatient	Self-treatment	Outpatient	Inpatient	Self-treatment	Outpatient	Inpatient	Self-treatment
**Demographics**									
(Age and gender)	1.61%	5.27%	1.85%	1.59%	4.54%	7.45%	3.69%	10.77%	2.62%
**Circumstances**									
Urban/rural residence	5.57%	10.45%	6.11%	14.00%	13.22%	13.74%	14.59%	30.55%	6.57%
Basic medical insurance	0.10%	0.64%	0.05%	1.98%	0.71%	0.87%	0.01%	0.13%	0.09%
OOP ratio	40.68%	5.98%	7.00%	43.71%	15.24%	8.18%	40.94%	14.94%	13.42%
Distance	5.88%	17.33%	9.71%	22.31%	2.67%	8.62%	16.93%	9.60%	1.65%
Education	2.26%	4.66%	0.50%	5.53%	18.46%	2.44%	0.01%	0.65%	0.50%
Work Status	5.12%	24.40%	7.05%	0.93%	6.00%	5.16%	7.84%	19.26%	13.40%
Income	2.16%	9.10%	1.83%	0.86%	21.20%	0.44%	0.03%	2.53%	0.17%
**Total**	**61.77%**	**72.56%**	**32.25%**	**89.32%**	**77.50%**	**39.45%**	**80.35%**	**77.66%**	**35.80%**
**Efforts**									
Health status (Self-reported health status and chronic disease)	27.53%	12.99%	59.97%	2.98%	0.63%	43.58%	12.70%	8.94%	59.16%
Supplemental medical insurance	0.40%	2.65%	0.14%	2.72%	13.89%	0.02%	1.14%	1.29%	0.38%
Physical examination	8.68%	6.51%	5.80%	3.40%	3.44%	9.50%	2.12%	1.35%	2.03%
**Total**	**36.61%**	**22.15%**	**65.91%**	**9.10%**	**17.96%**	**53.10%**	**15.96%**	**11.58%**	**61.57%**

Note: Demographics include age and gender. Health status includes self-reported health status and chronic disease

With regard to the specific contributors of circumstances, the OOP ratio was the most explanatory factor for the IOp of outpatient expenditures, with a contribution of 40.68%–43.71% from 2013 to 2018, which was much higher than other circumstance factors. Distance (5.88%–22.31%), residence (5.57%–14.59%), and work status (0.93%–7.84%) were also factors that led to the IOp of outpatient expenditures, and their contributions were all increased. From 2013 to 2018, residence area highly explained the IOp of inpatient service utilization and its contribution increased over time (from 10.45% in 2013 to 30.55% in 2018). Work status (6.00%–24.40%), OOP ratio (5.98%–15.24%) and income (2.53%–21.20%) are also the main contributors to the IOp of inpatient service. Among all circumstance factors, the contributions of OOP ratio (7.00%–13.42%), residence (6.11%–13.74%), and work status (5.16%–13.40%) to the IOp of self-treatment expenditures were relatively slight but all increased from 2013 to 2018. The contribution of basic medical insurance to IOp of three health services utilization remained low in all waves.

## Discussion

At present, China is under the realistic background of huge differences in the utilization of medical services and the policy background of the government constantly promoting the equalization of medical services [29]. Deepening the research on the unequal utilization of medical services is conducive to formulate more reasonable health policies. Based on Roemer's framework of equality of opportunity, we think that the imbalanced utilization caused by individual health needs is reasonable. Actually, there is unreasonable inequality in health service utilization, but the extent of inequality and its contributors are less discussed. Therefore, using national representative survey data from CHARLS, we investigated the related factors of the utilization of outpatient, inpatient, and self-treatment services among Chinese middle-aged and elderly people; quantified the absolute IOp and relative IOp of the utilization of these 3 kinds of health services; and estimated the contributors of IOp. This study innovatively expanded measures of IOp and its decomposition into the area of health service utilization among middle-aged and elderly adults and provided new evidence for improving health service equity in China.

Here, we found that the utilization of outpatient, inpatient, and self-treatment services among Chinese middle-aged and elderly people improved from 2013 to 2018, but differences in health service utilization among individuals still existed. The empirical results showed that residence area, OOP ratio, medical distance, work status in circumstance factors and physical examination in effort factors are important explanatory variables of the 3 types of health service utilization among middle-aged and elderly people (Table 3), which is mirrored by previous studies [32, 50, 51].

The IOp of outpatient care, inpatient care, and self-treatment utilization among the middle-aged and elderly overall declined over time (Table 4): the IOp ratio of outpatient care decreased from 36.85% to 34.06%, that of inpatient care decreased from 50.18% to 36.33%, and that of self-treatment decreased from 38.73% to 36.86%, respectively. This trend may be due to the great progress made in China's health care reform in recent years, such as the construction of a 3-tier health service network and the expansion of basic medical insurance coverage, which have increased the availability and affordability of health services. However, the inequality of opportunity is still serious. The absolute IOp of self-treatment is the highest among the 3 medical services. Self-treatment is usually considered a fast, accessible, and more convenient method of health service utilization among middle-aged and elderly people. However, the cost of self-treatment is usually not covered by medical insurance in China, which may reduce the willingness of middle-aged and elderly people to utilize self-treatment. Self-treatment is more sensitive to circumstance factors, such as social health insurance and income, which may lead to significant IOp. Therefore, medical insurance policies should further expand their coverage and increase the reimbursement rate for self-treatment utilization so as to reduce the IOp. The highest ratio of IOp in total inequality is in inpatient services, consistent with the results of Zhang et al. [28]. As we all know, inpatient service may have the highest cost of all the health services utilization, while groups with poor insurance benefits or low income may be prevented from using such health care, which indicating that hospitalization may have more unreasonable inequalities than the other 2 health service types, and it is more difficult to improve through self-efforts. Therefore, policy measures should pay more attention to the circumstance factors of inpatient care to alleviate the IOp of health service utilization.

Shapley decomposition revealed that the contribution of circumstance factors to the unequal utilization of various health services is gradually expanding; however, the contribution of individual effort factors is gradually weakening. Among circumstance factors, OOP ratio contributed the most to the IOp of outpatient care. This may indicate that differences in individual co-payment levels may account for much of the inequality in outpatient care, which is also confirmed by prior literature [52, 53]. At present, China's basic medical insurance system still does not provide a high reimbursement level for outpatient care. It is universally accepted that a reasonable OOP proportion is 30%–40% for health expenditures [54], but, as of 2018, the OOP proportion of outpatient care for middle-aged and elderly people in China was 86.79% according to this study. Additionally, there are still dramatical discrepancies in security level between different insurance insurances in China—for example, the reimbursement level of resident medical insurance is lower than that of employee medical insurance [35, 38], which may lead to different IOp of health service utilization for middle-aged and elderly people having different medical insurances. Hence, the government should further optimize medical insurance policies to increase the security level of outpatient service utilization and narrow the reimbursement gap between different medical insurances.

In contrast, some socioeconomic variables in circumstance factors, such as work status and income, have limited contributions to the IOp of outpatient and self-treatment but a great impact on the IOp of inpatient service, respectively. This suggests that occupational and income factors play an important role in inpatient utilization, and these differences in socioeconomic status greatly contribute to the IOp of hospitalization utilization. Inpatient care utilization is sensitive to individuals' socioeconomic factors due to the higher costs associated with it. The middle-aged and elderly with informal jobs or lower incomes are always at risk of being prevented from taking advantage of inpatient utilization. Fortunately, the contribution of work status and income to the IOp of inpatient care utilization is decreasing according to our findings. Policy efforts should continue to focus on increasing the income level or providing medical subsidies for middle-aged and elderly people with low socioeconomic status to reduce the inequality of opportunities [33].

The change of medical distance's contribution to IOp of the three types of health service utilization also deserves our attention. Medical distance's contribution to the IOp of hospitalization and self-treatment decreased from 2013 to 2018, which may reflect the recent implementation effect of the new medical reform policy in China, such as timely settlement of medical insurance in non-local treatment [55], which makes it more convenient for residents to obtain inpatient care and self-treatment services across regions. It is also worth noting that residents' medical distance is getting farther and farther from 2013 to 2018, and the medical distance has aggravated the IOp in outpatient service utilization. One possible explanation is that health resources in China are still scarce and scattered, which reflects the inequality of the geographical distribution of health resources in China, and similar findings also exist in the research of other scholars [56]. Therefore, it is necessary to increase the investment in areas with weak health resources according to distance, demographic structure, transportation convenience, and medical needs to ensure equitable access to health services [57].

Our study also confirms the dramatical influence of residence area on the unequal utilization of health services, which is consistent with existing studies [34, 51, 52], suggesting that there is still a considerable difference in health service utilization between urban and rural middle-aged and elderly people. The possible reason for this phenomenon is that, due to the unbalanced social and economic development between urban and rural areas in China, the socioeconomic status of middle-aged and elderly people in rural areas is more vulnerable than that of those in urban areas, and high medical expenditures are more likely to cause them to become impoverished, resulting in IOp of health service utilization. Although China implemented the policy of universal coverage of basic medical insurance in 2016, there is still a big gap between urban and rural areas in terms of medical service coverage, medical insurance financing level, resource allocation and policy implementation effect in China at present, resulting in differentiated medical supply between urban and rural areas [52]. Therefore, policies should still be tilted towards rural middle-aged and elderly people, such as optimizing the medical security system and regional medical resource allocation level [58] to improve the accessibility of rural medical services. In addition, measures such as strengthening health education and improving employment to narrow the gap between the social and economic status of middle-aged and elderly people in urban and rural areas should also be taken, so as to reduce the IOp of health service utilization caused by residential areas.

There are still some limitations that need to be acknowledged. We only used expenditure variables to measure personal health service utilization, without addressing more detailed health care demands or health-service quality variables, which may underestimate health service utilization to some extent. Besides, the expenditure data we used were self-reported by respondents, which may have recall bias and may not reflect the true health service utilization. Furthermore, there may be more circumstance factors leading to the IOp of health service utilization than those listed in this study. However, we could not include all related factors due to questionnaire limitations. Further studies will further explore the inequality caused by price or supply factors of medical services.

## Conclusion

Our research found that the absolute IOp of outpatient, inpatient, and self-treatment health service utilization among China's middle-aged and elderly people declined from 2013 to 2018, but the contribution of circumstance factors to the total inequality was still significant. The OOP ratio, residence area, work status, and income were the main contributors to the IOp of health service utilization, indicating that policies should focus on further increasing the security level and narrowing the reimbursement gap between different basic medical insurances to alleviate the IOp. In addition, policy efforts should pay more attention to the middle-aged and elderly residents in rural areas, further optimize health resource allocation, improve the social security systems, and narrow the socioeconomic gap between urban and rural areas in China.

## Data Availability

The datasets used in this study are publicly available from China Health and Retirement Longitudinal Study, http://charls.pku.edu.cn.

## Abbreviations

IOp: Inequality of Opportunity
CHARLS: China Health and Retirement Longitudinal Survey
PPS: Probability Proportional to Size
OOP: Out-of-pocket
OLS: Ordinary least squares
